# Clinical risk factors and social needs of 30-day readmission among patients with diabetes: A retrospective study of the Deep South

**DOI:** 10.3389/fcdhc.2022.1050579

**Published:** 2022-10-26

**Authors:** Cassidi C. McDaniel, Chiahung Chou

**Affiliations:** ^1^ Department of Health Outcomes Research and Policy, Harrison College of Pharmacy, Auburn University, Auburn, AL, United States; ^2^ Department of Medical Research, China Medical University Hospital, Taichung, Taiwan

**Keywords:** diabetes, readmission, transitions of care, risk factor, social need, Deep South

## Abstract

**Introduction:**

Evidence is needed for 30-day readmission risk factors (clinical factors and social needs) among patients with diabetes in the Deep South. To address this need, our objectives were to identify risk factors associated with 30-day readmissions among this population and determine the added predictive value of considering social needs.

**Methods:**

This retrospective cohort study utilized electronic health records from an urban health system in the Southeastern U.S. The unit of analysis was index hospitalization with a 30-day washout period. The index hospitalizations were preceded by a 6-month pre-index period to capture risk factors (including social needs), and hospitalizations were followed 30 days post-discharge to evaluate all-cause readmissions (1=readmission; 0=no readmission). We performed unadjusted (chi-square and student’s t-test, where applicable) and adjusted analyses (multiple logistic regression) to predict 30-day readmissions.

**Results:**

A total of 26,332 adults were retained in the study population. Eligible patients contributed a total of 42,126 index hospitalizations, and the readmission rate was 15.21%. Risk factors associated with 30-day readmissions included demographics (e.g., age, race/ethnicity, insurance), characteristics of hospitalizations (e.g., admission type, discharge status, length of stay), labs and vitals (e.g., highest and lowest blood glucose measurements, systolic and diastolic blood pressure), co-existing chronic conditions, and preadmission antihyperglycemic medication use. In univariate analyses of social needs, activities of daily living (p<0.001), alcohol use (p<0.001), substance use (p=0.002), smoking/tobacco use (p<0.001), employment status (p<0.001), housing stability (p<0.001), and social support (p=0.043) were significantly associated with readmission status. In the sensitivity analysis, former alcohol use was significantly associated with higher odds of readmission compared to no alcohol use [aOR (95% CI): 1.121 (1.008-1.247)].

**Conclusions:**

Clinical assessment of readmission risk in the Deep South should consider patients’ demographics, characteristics of hospitalizations, labs, vitals, co-existing chronic conditions, preadmission antihyperglycemic medication use, and social need (i.e., former alcohol use). Factors associated with readmission risk can help pharmacists and other healthcare providers identify high-risk patient groups for all-cause 30-day readmissions during transitions of care. Further research is needed about the influence of social needs on readmissions among populations with diabetes to understand the potential clinical utility of incorporating social needs into clinical services.

## Introduction

According to the International Diabetes Federation as of 2021, diabetes affects 536.6 million people across the world (1). Diabetes is most prevalent in high-income countries (HIC) at 11.1% compared to middle-income (10.8%) and low-income (5.5%) countries (1). Problematic increases in the prevalence of diabetes are expected across the world in the coming decades, and predictions reveal that low- and middle-income countries (LMIC) will account for 94% of the increased prevalence by 2045 (1). The World Health Organization’s Global Report on Diabetes highlights the burden of diabetes due to complications, such as vision impairment, kidney problems, cardiovascular disease, and lower extremity amputations (2). Additional burdens resulting from complications of diabetes or other co-existing chronic conditions might include hospitalizations and subsequent readmissions, which are the focus of this report.

Populations with diabetes are at risk of experiencing burdensome hospitalizations. Diabetes and related complications have been estimated to be the fifth leading reason for hospital admissions in the U. S. (3). The process of patients receiving hospital care, being discharged from hospital care, and returning to home care is known as a ‘transition of care’ (4). Transitions of care have been referred to as “vulnerable exchange points that contribute to unnecessarily high rates of health services use and health care spending” that “expose chronically ill people to lapses in quality and safety” (4). This point is particularly true for populations with diabetes, who are highly susceptible to readmissions within the next 30 days after hospital discharge (5, 6). This 30-day period is a commonly used target indicator for risk standardization by the Centers for Medicare and Medicaid Services (CMS) (7). Patients with diabetes face significantly higher all-cause 30-day readmission rates than those without diabetes (24.3% versus 17.7%, respectively) (8) and longer hospital stays (9). These frequent and longer duration hospitalizations constitute a sizeable economic burden globally for patients with diabetes (2). Reducing 30-day readmission rates is essential to decreasing medical expenditures (10). Overall, readmissions among populations with diabetes are an important public health issue in diabetes care.

In efforts to inform evidence for reducing readmissions, prior research studied risk factors associated with readmissions for patients with diabetes, but evidence gaps remain. In 2020, a systematic review and meta-analysis pooled findings across 18 studies to estimate the influence of risk factors for 30-day readmissions among populations with diabetes (11). The risk was higher based on male gender, older age, non-White race, Medicare and Medicaid insurance coverage, the presence of comorbidities, longer length of stay, and use of insulin (11). In accordance with findings from this systematic review, literature on risk factors has focused mainly on patient demographics and clinical data elements from electronic health records (EHRs) to predict readmissions for populations with diabetes (9, 11–16). Also, the EHRs used in these investigations mostly covered populations in the Northeast region of the U.S., so the generalizability to people living in the Deep South is questionable. Additionally, looking outside the scope of demographics and healthcare alone is needed given the recognized impact of non-healthcare factors, like social determinants of health and behaviors, on diabetes care, management, and outcomes (17).

Social and behavioral factors are now widely recognized to influence health outcomes. It is essential to consider these factors (which we will refer to as ‘social needs’) during the investigation of readmissions when these patient-reported measures are captured through data integration in EHRs (18). CMS defines social needs as “individual-level, adverse social conditions that can negatively impact a person’s health or health care” (19). Social needs, such as homelessness, substance use, and challenges affording basic needs (e.g., food, clothing, utilities) or healthcare, have been linked with preventable 30-day readmissions (20). Further, a recent national investigation revealed an increased risk of readmission with a higher number of unmet social needs; the readmission rate more than doubled between having no social needs (11.5%) versus one social need (27.0%) (21). These prior investigations of social needs were among the general adult population (19–21), so the impact of social needs on readmission for people with diabetes remains unclear.

Patients with diabetes living in the Deep South have not been a population of focus for the literature studying readmission risk or transitions of care, despite the disproportionately higher diabetes prevalence in this area (22). Thus, our study seeks to expand and generalize prior research on readmission risk factors for patients with diabetes to the Deep South. The novelty of our approach includes expanding the risk factors considered to include social needs, along with differentiation of readmission status by diabetes type (type 1 versus type 2 diabetes). Our objectives were to identify risk factors associated with all-cause 30-day readmissions among patients with diabetes in the Deep South and determine whether social needs added value in predicting all-cause 30-day readmissions. Findings of risk factors associated with readmissions will apply to the unique needs of patients with diabetes in the Deep South. Our definition of the Deep South includes states such as Alabama, Georgia, Louisiana, Mississippi, South Carolina, and Tennessee (23, 24), and findings will be represented by a population of patients with diabetes in Alabama.

## Materials and methods

### Study design and data source

This retrospective cohort study used EHRs from an urban health system in the Southeastern U.S. from January 1, 2016 through October 1, 2020. Data from the EHRs were generated through routine clinical practice, and the data were not collected for research purposes. The EHRs were de-identified and extracted to be used for secondary research in this study. Because the pre-existing, de-identified EHRs were used, this research did not involve any interaction with patients. The study protocol was reviewed and approved by the Auburn University Institutional Review Board for the Protection of Human Subjects in Research (IRB) under the exempt review application process.

### Study population

**Figure 1:**
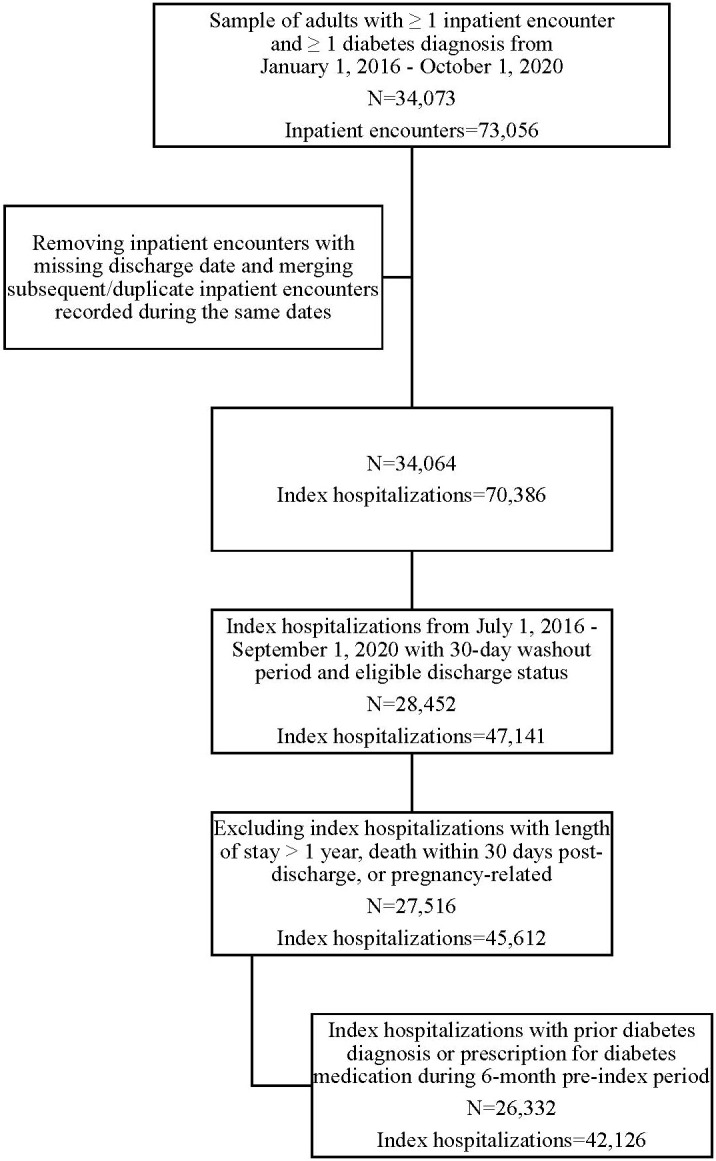
Flow diagram for cohort identification.

Patients eligible for inclusion were adults (≥18 years old at the time of hospital admission) diagnosed with diabetes (type 1 or type 2) before an inpatient hospitalization. Diagnosis of diabetes was identified through at least one diabetes diagnosis code before the index hospitalization (ICD-9-CM or ICD-10-CM from the Chronic Conditions Warehouse (25) or SNOMED code). People without a diabetes diagnosis were included and assumed to have diabetes if a prescription for diabetes medication was ordered during the 6-month period before hospitalization (13, 15). Patients with diagnosis codes for gestational diabetes were excluded. The cohort flow diagram (**Figure 1**) depicts how the original sample of adults with diabetes and ≥ one inpatient encounter was selected from the EHRs. From this original sample, we made further restrictions to identify eligible index hospitalizations following the eligibility criteria outlined in Outcomes section below. When identifying the eligible study population, we did not consider patients’ socioeconomic status within the sampling strategy because data for socioeconomics were not available in the EHRs.

### Outcomes

The primary outcome was all-cause 30-day readmission, which was operationalized dichotomously as 1=readmission versus 0=no readmission. We focused on all-cause 30-day readmissions, as opposed to diabetes-related readmissions, to reflect the overall healthcare experiences during readmissions for patients with diabetes. We used index hospitalization as the unit of analysis so that each person could have multiple index hospitalizations. We followed each index hospitalization for 30 days after discharge to evaluate readmission status. Subsequent hospitalizations that were recorded during the same or overlapping time periods (e.g., the same discharge date) were counted as the same hospitalization to avoid duplicate counting of hospitalizations (13, 26).

We required that index hospitalizations be preceded by a 6-month pre-index period to capture baseline risk factors (12). We also required that index hospitalizations have a 30-day washout period (with no documented inpatient hospitalization) before the admission date to ensure the hospitalization was an initial admission and not a readmission itself (12). We excluded index hospitalizations that were pregnancy-related [following prior literature of pregnancy-related diagnosis codes (27)] or had a length of stay longer than one year (26). We also excluded hospitalizations with missing discharge dates or hospitalizations discharged to hospital transfer or unknown/missing discharge status (13, 15). Hospitalizations discharged to hospice were also excluded to remove patients receiving ‘end-of-life care’ due to the variability of readmissions after discharge to hospice (28). Lastly, we excluded hospitalizations with documented death during hospitalization (13, 15) or death within 30 days post-discharge (when readmission did not precede the death date).

### Risk factors

The selection and inclusion of specific risk factors were driven by prior literature that identified associations with 30-day readmissions among patients with diabetes. The research conducted previously indicates that various factors may impact readmission risk in diabetes, such as demographics, labs, medication use, healthcare utilization, other chronic conditions, etc. (9, 11–15, 26). We incorporated findings of readmission risk factors from the foundational works of Rubin et al. through validation of the Diabetes Early Readmission Risk Indicator (DERRI™) (14, 15) and Karunakaran et al. through expansion of the DERRI™ to include additional pre- and post-discharge risk factors (13). However, we were limited to including risk factors available through data elements in the EHRs. Details about the measurement of all risk factors can be found in the **Supplemental Table** (see **Supplementary Material**).

Prior literature guided our operationalization of risk factors (9, 11–15, 26). We differentiated between type 1 and type 2 diabetes based on diagnosis codes any time before the index hospitalization. Diabetes type was coded as unknown when no diagnosis code was present, but a prescription for a diabetes medication was filled during the 6-month pre-index period. Preadmission prescriptions for diabetes medications were classified into the following medication classes: insulin, metformin, sulfonylureas, thiazolidinediones (TZD), dipeptidyl peptidase 4 (DPP-4) inhibitors, sodium-glucose cotransporter-2 (SGLT2) inhibitors, glucagon-like peptide-1 (GLP-1) receptor agonists, and others (meglitinides, α-glucosidase inhibitors, amylin analog, cycloset, bile acid sequestrants). Prior research found extreme blood glucose measures [i.e., high (>180 mg/dL) and low (<70 mg/dL)] to be associated with readmissions (13), so we retained the highest and lowest blood glucose value during the index hospitalization to represent proxy variables for hyperglycemia and hypoglycemia, respectively. For labs (HbA1c, albumin, creatinine, hematocrit, white blood cell count, potassium, sodium), body mass index (BMI), and blood pressure, we retained the record nearest to the index hospitalization admission date (15). HbA1c was capped at 3.5% and 18.5% in the EHRs. Implausible values for other labs and blood pressure were excluded following cut-offs recommended by Estiri et al. (29), except for creatinine [<0.1 mg/dL or >15 mg/dL was used (30)] and hematocrit (observations outside of six standard deviations were removed as an alternative approach used by Estiri). We also excluded implausible values for BMI [<10 kg/m^2^ or >100 kg/m^2^ (30)]. Vitals, including BMI, systolic blood pressure, and diastolic blood pressure, were converted to categorical variables based on clinical cut-points from the National Heart, Lung, and Blood Institute (31) and the American College of Cardiology/American Heart Association (32). We captured comorbidities using the Charlson Comorbidity Index (CCI) (33).

Risk factors for the index hospitalization were included, such as length of stay, admission type (i.e., emergency, elective, urgent, trauma), and discharge status (i.e., home, home health, skilled nursing facility, against medical advice, other). Discharge statuses of ‘other’ included discharges to court/law enforcement, custodial care or support, federal facilities, psychiatric facilities, short-term facilities, and other facilities. We captured macrovascular and microvascular complications using ICD-9-CM codes from Karunakaran et al. (13), and we translated these to ICD-10-CM codes using the following resources: icd9data.com, icd10data.com, and Glasheen et al. (33). Social needs were available through structured data obtained from patients’ self-reported measures in the EHRs; the social needs were descriptive measures, not validated questionnaires. We followed the definition and conceptualization from CMS to include the following 20 social needs (19): activities of daily living, feeling unsafe at home, alcohol use, substance use, smoking/tobacco use, e-cigs/vaping device use, household characteristics (i.e., abuse, alcohol abuse, substance abuse, smoking), employment, work activity level, education, financial security, housing stability, living situation, social support, feeling stressed, stressors, and physical activity. We retained the social needs response from the record nearest to the index hospitalization admission date. Some social needs were not included in the analyses due to high rates of missingness in the data source. For instance, food security was not included because responses were missing for >99% of index hospitalization records.

### Statistical analysis

We stratified index hospitalizations by all-cause 30-day readmission status. For univariate analyses, chi-square and t-tests (where applicable) compared risk factors for index hospitalizations with versus without 30-day readmission. Risk factors associated with 30-day readmission status at the p<0.10 level were included in the adjusted analyses (13). For adjusted analyses, we used logistic regression with generalized estimating equations (GEE) to account for the correlation within individuals because one individual could have multiple index hospitalizations (13–15). We employed a multivariable logistic regression model with GEE for the adjusted analysis and reported adjusted odds ratios (aOR) and 95% confidence intervals (95% CIs) for risk factors to demonstrate their relationship with 30-day readmission status. We did not include macrovascular complications in the adjusted regression model because this information was already captured in the CCI calculation, while microvascular complications were retained in the model.

We used chi-square tests to compare social needs for index hospitalizations with versus without 30-day readmission. We evaluated whether social needs added value in predicting all-cause 30-day readmissions by comparing a multivariable logistic regression model without social needs to the model with social needs. After controlling for all covariates in the baseline regression model, a significant association between the newly added variables (i.e., social needs) was considered to add predictive value (34). We used SAS, version 9.4 (SAS Institute, Cary, NC) for data analyses.

#### Handling of missing data

We treated missing EHRs data to be missing at random based on the possibility that the documentation of clinical records or reporting of social needs could be related to patients’ healthcare utilization. We imputed missing data using multiple imputations by chained equations (MICE), also known as fully conditional specification, which has been recommended as a valid method to handle missing EHR data (35). During the imputation of missing data, we followed recommendations from Wells et al. to perform imputation while considering variables representing healthcare use (prior hospitalization, follow-up appointment post-discharge), diabetes severity (HbA1c), comorbidities (CCI score), socio-economic status (insurance type), and the outcome (30-day readmission) (35). The levels of missingness were highest for social needs variables (reported in **Table 2**), so we performed a sensitivity analysis to remove social needs with high levels of missingness (≥70%) from the regression model.

## Results

Among the original sample of 34,073 adults with diabetes and one or more inpatient hospitalization, 26,332 adults were retained after applying eligibility criteria (**Figure 1**). Of the 42,126 index hospitalizations, 6407 (15.21%) were followed by all-cause 30-day readmissions. Among those readmitted, the average time to readmission was approximately 13 days (Mean (SD)=13.34 (8.41), Median=12.00, Min=1, Max=30).

### Characteristics of study population

Personal characteristics, including race/ethnicity, marital status, and insurance coverage, were associated with readmission status (p<0.001), while age and gender were not significantly associated with readmission status. The year of discharge was not significant in univariate analysis (p=0.862). The distribution of other risk factors with readmission status can be found in **Table 1**.

**Table 1:** Demographic characteristics and risk factors of index hospitalizations.

Characteristics/Risk factors	Readmission status N=26,332 Hospitalizations (Hosp.)=42,126		Missing %	p-value
	Readmission Hosp. = 6407	No readmission Hosp. = 35,719		
Age, Mean (SD)	59.98 (14.52)	60.33 (14.57)	0	0.080
Race/Ethnicity, N (%)			0	<0.001
Non-Hispanic White	3281 (51.21)	18,263 (51.13)		
Non-Hispanic Black	2727 (42.56)	14,751 (41.30)		
Non-Hispanic Asian	143 (2.23)	741 (2.07)		
Hispanic	89 (1.39)	545 (1.53)		
Other/unknown	167 (2.61)	1419 (3.97)		
Gender, N (%)			0	0.504
Female	3139 (48.99)	17,662 (49.45)		
Male	3268 (51.01)	18,057 (50.55)		
Marital status, N (%)			<1	<0.001
Married/Life partner	2691 (42.09)	15,804 (44.72)		
Single	1866 (29.18)	10,121 (28.64)		
Widowed	812 (12.70)	4423 (12.52)		
Divorced	859 (13.43)	4197 (11.88)		
Separated	166 (2.60)	792 (2.24)		
Insurance, N (%)			0	<0.001
Medicare	3970 (61.96)	20,866 (58.42)		
Private	1063 (16.59)	7215 (20.20)		
Medicaid	859 (13.41)	3815 (10.68)		
Self-pay	235 (3.67)	1788 (5.01)		
Other/unknown	134 (2.09)	1011 (2.83)		
VA/Tricare	81 (1.26)	629 (1.76)		
Indigent/charity care	65 (1.01)	395 (1.11)		
Diabetes type, N (%)			0	<0.001
Type 2	5939 (92.70)	33,600 (94.07)		
Type 1	366 (5.71)	1850 (5.18)		
Unknown	102 (1.59)	269 (0.75)		
Admission type, N (%)			<0.1	<0.001
Emergency	4304 (67.18)	21,875 (61.27)		
Elective	1040 (16.23)	8220 (23.02)		
Urgent	974 (15.20)	4599 (12.88)		
Trauma	89 (1.39)	1007 (2.82)		
Discharge status, N (%)			0	<0.001
Home	3448 (53.82)	22,186 (62.11)		
Home health	2068 (32.28)	9593 (26.86)		
Skilled nursing facility	732 (11.43)	3153 (8.83)		
Against medical advice	57 (0.89)	245 (0.69)		
Other	102 (1.59)	542 (1.52)		
Body mass index, N (%)			11	<0.001
Underweight (<18.5 kg/m^2^)	185 (3.18)	705 (2.23)		
Healthy weight (18.5-24.9 kg/m^2^)	1333 (22.93)	6043 (19.15)		
Overweight (25.0-29.9 kg/m^2^)	1581 (27.20)	8630 (27.34)		
Obese (≥30 kg/m^2^)	2714 (46.69)	16,183 (51.28)		
Systolic blood pressure, N (%)			0	<0.001
Normal/elevated (<130 mmHg)	2968 (46.32)	14,953 (41.86)		
Stage 1 HTN (130-139 mmHg)	896 (13.98)	5291 (14.81)		
Stage 2 HTN (≥140 mmHg)	2543 (39.69)	15,475 (43.32)		
Diastolic blood pressure, N (%)			0	<0.001
Normal (<80 mmHg)	4257 (66.44)	22,863 (64.01)		
Stage 1 HTN (80-89 mmHg)	1138 (17.76)	7255 (20.31)		
Stage 2 HTN (≥90 mmHg)	1012 (15.80)	5601 (15.68)		
HbA1c (%), Mean (SD)	7.74 (2.37)	7.84 (2.42)	27	0.009
Albumin (gm/dL), Mean (SD)	3.48 (0.64)	3.64 (0.60)	19	<0.001
Creatinine (mg/dL), Mean (SD)	2.05 (2.32)	1.75 (2.02)	<1	<0.001
Highest blood glucose (mg/dL), Mean (SD)	283.80 (133.1)	270.30 (127.6)	<1	<0.001
Lowest blood glucose (mg/dL), Mean (SD)	94.14 (34.81)	100.70 (36.43)	<1	<0.001
Hematocrit (%), Mean (SD)	33.91 (6.88)	35.63 (6.64)	<1	<0.001
White blood cell count (10^3^/cmm), Mean (SD)	9.98 (10.02)	9.74 (6.19)	<1	0.062
Potassium (mMol/L), Mean (SD)	4.19 (0.67)	4.16 (0.63)	<1	0.006
Sodium (mMol/L), Mean (SD)	136.30 (4.43)	136.60 (4.16)	<1	<0.001
Length of stay (days), Mean (SD)	8.16 (10.53)	6.42 (9.34)	0	<0.001
Charlson Comorbidity Index, Mean (SD)	6.95 (4.03)	5.38 (3.71)	0	<0.001
Macrovascular complications, N (%)			0	<0.001
0	2025 (31.61)	13,733 (38.45)		
1	1678 (26.19)	10,121 (28.34)		
2	1775 (27.70)	8307 (23.26)		
3	814 (12.70)	3177 (8.89)		
4	115 (1.79)	381 (1.07)		
Microvascular complications, N (%)			0	<0.001
0	3037 (47.40)	20,455 (57.27)		
1	2241 (34.98)	10,828 (30.31)		
2	870 (13.58)	3521 (9.86)		
3	259 (4.04)	915 (2.56)		
Anemia diagnosis, N (%)			0	<0.001
No	2476 (38.65)	19,055 (53.35)		
Yes	3931 (61.35)	16,664 (46.65)		
Preadmission insulin use, N (%)			0	<0.001
No	3208 (50.07)	22,238 (62.26)		
Yes	3199 (49.93)	13,481 (37.74)		
Preadmission metformin use, N (%)			0	<0.001
No	5551 (86.64)	29,867 (83.62)		
Yes	856 (13.36)	5852 (16.38)		
Preadmission sulfonylurea use, N (%)			0	0.210
No	5973 (93.23)	33,143 (92.79)		
Yes	434 (6.77)	2576 (7.21)		
Preadmission GLP-1 use, N (%)			0	0.020
No	6284 (98.08)	34,863 (97.60)		
Yes	123 (1.92)	856 (2.40)		
Preadmission DPP-4 use, N (%)			0	0.653
No	6236 (97.33)	34,730 (97.23)		
Yes	171 (2.67)	989 (2.77)		
Preadmission SGLT2 use, N (%)			0	0.060
No	6337 (98.91)	35,224 (98.61)		
Yes	70 (1.09)	495 (1.39)		
Preadmission TZD use, N (%)			0	0.011
No	6373 (99.47)	35,421 (99.17)		
Yes	34 (0.53)	298 (0.83)		
Preadmission other diabetes medications*, N (%)			0	0.101
No	6365 (99.34)	35,542 (99.50)		
Yes	42 (0.66)	177 (0.50)		
Prior admission within 90 days of index hospital admission, N (%)			0	<0.001
No	5712 (89.15)	34,026 (95.26)		
Yes	695 (10.85)	1693 (4.74)		
Discharge status of most recent hospital stay within last year, N (%)			0	<0.001
No hospitalization within past year	4638 (72.39)	30,449 (85.25)		
Home	925 (14.44)	2847 (7.97)		
Home health	540 (8.43)	1483 (4.15)		
Skilled nursing facility	136 (2.12)	364 (1.02)		
Against medical advice	20 (0.31)	42 (0.12)		
Other	148 (2.31)	534 (1.50)		
Follow-up appointment after discharge, N (%)			0	0.001
Yes	4144 (64.68)	22,331 (62.52)		
No	2263 (35.32)	13,388 (37.48)		
Discharge year, N (%)			0	0.862
2016	671 (10.47)	3782 (10.59)		
2017	1522 (23.76)	8322 (23.30)		
2018	1571 (24.52)	8751 (24.50)		
2019	1593 (24.86)	9075 (25.41)		
2020	1050 (16.39)	5789 (16.21)		

*Other diabetes medications include meglitinides, α-glucosidase inhibitors, amylin analog, cycloset, and bile acid sequestrants.

VA, Veterans Administration; HTN, hypertension; GLP-1, glucagon-like peptide-1 receptor agonists; TZD, thiazolidinediones; DPP-4, dipeptidyl peptidase 4 inhibitors; SGLT2, sodium–glucose cotransporter-2 inhibitors.

### Distribution of social needs by readmission status

The distributions of social needs by readmission status are presented in **Table 2**. For activities of daily living, readmission was associated with higher distributions of needing some help or being dependent on others compared to no readmission (p<0.001; see **Table 2** for frequencies). Readmission was associated with higher distributions of former alcohol use (p<0.001), former substance use (p=0.002), and former smoking/tobacco use (p<0.001) compared to no readmission. Readmission was associated with higher distributions of disabled employment status and lower distributions of employed status compared to no readmission (p<0.001). Unstable housing was more frequent among the readmission group than the no-readmission group (p<0.001), and social support was significantly associated with readmission status (p=0.043). Social needs demonstrating an insignificant relationship with readmission status included feeling unsafe at home, household characteristics (i.e., abuse, alcohol abuse, substance abuse, smoking), using vaping devices, work activity level, education level, financial security, living situation, feeling stressed, types of stressors, and participating in physical activity (p≥0.05 for all).

**Table 2:** Social needs of index hospitalizations.

Social needs	Readmission status N=26,332 Hospitalizations (Hosp.)=42,126		Missing %	p-value
	Readmission Hosp. = 6407 Hosp. (%)	No readmission Hosp. = 35,719 Hosp. (%)		
Activities of daily living			80	<0.001
Independent	1038 (72.13)	5548 (77.10)		
Needs some help	317 (22.03)	1345 (18.69)		
Dependent	84 (5.84)	303 (4.21)		
Feels unsafe at home			70	0.340
No	2005 (96.81)	10,278 (96.39)		
Yes	66 (3.19)	385 (3.61)		
Household abuse			45	0.633
No	3586 (99.17)	19,464 (99.09)		
Yes	30 (0.83)	179 (0.91)		
Alcohol use			44	<0.001
Current	628 (17.41)	4496 (22.50)		
Former	586 (16.24)	2502 (12.52)		
None	2280 (63.19)	12,329 (61.71)		
Within the past year	114 (3.16)	651 (3.26)		
Substance use			45	0.002
Current	193 (5.41)	1143 (5.80)		
Former	255 (7.15)	1123 (5.70)		
None	3078 (86.34)	17,275 (87.68)		
Within the past year	39 (1.09)	162 (0.82)		
Smoking/tobacco use			40	<0.001
Current smoker	743 (19.02)	4260 (19.79)		
Former smoker	1507 (38.58)	7582 (35.22)		
Never smoked	1656 (42.40)	9685 (44.99)		
E-cigs/vaping device use			90	0.542
No	649 (96.43)	3543 (96.88)		
Yes	24 (3.57)	114 (3.12)		
Household alcohol abuse			85	0.955
No	1019 (96.31)	4909 (96.35)		
Yes	39 (3.69)	186 (3.65)		
Household substance abuse			86	0.688
No	1007 (95.90)	4834 (95.63)		
Yes	43 (4.10)	221 (4.37)		
Household smoking			86	0.379
No	771 (73.92)	3782 (75.22)		
Yes	272 (26.08)	1246 (24.78)		
Employment status			63	<0.001
Retired	853 (35.36)	4805 (36.46)		
Disabled	890 (36.90)	3927 (29.80)		
Employed	333 (13.81)	2515 (19.08)		
Unemployed	292 (12.11)	1639 (12.44)		
Others (part time or student)	44 (1.82)	292 (2.22)		
Work activity level			96	0.702
Desk/office	90 (33.83)	452 (30.79)		
Heavy physical work	38 (14.29)	218 (14.85)		
Moderate physical work	74 (27.82)	403 (27.45)		
Occasional physical work	64 (24.06)	395 (26.91)		
Education level			93	0.681
High school or less than high school	220 (46.03)	1282 (48.88)		
Some college	157 (32.85)	815 (31.07)		
University degree	74 (15.48)	396 (15.10)		
Postgraduate	27 (5.65)	130 (4.96)		
Financial security			88	0.766
No	675 (79.98)	3340 (80.42)		
Yes	169 (20.02)	813 (19.58)		
Housing stability			54	<0.001
Home	2712 (91.19)	15,487 (93.35)		
Unstable housing	36 (1.21)	143 (0.86)		
Homeless	40 (1.34)	230 (1.39)		
Others	186 (6.25)	730 (4.40)		
Living situation			49	0.864
Lives with someone	2364 (74.60)	13,704 (74.45)		
Lives alone	805 (25.40)	4702 (25.55)		
Social support			86	0.043
Yes	907 (89.45)	4254 (87.14)		
No	107 (10.55)	628 (12.86)		
Feels stressed			92	0.480
No	410 (71.18)	1990 (69.70)		
Yes	166 (28.82)	865 (30.30)		
Stressors			98	0.083
Health-related stressor	89 (61.38)	464 (59.72)		
Finances	33 (22.76)	134 (17.25)		
Social stressor	23 (15.86)	179 (23.04)		
Physical activity			70	0.417
No	1213 (58.07)	6029 (57.10)		
Yes	876 (41.93)	4529 (42.90)		

### Likelihood of readmission from regression models

The adjusted odds for the multivariable logistic regression models without and with social needs are presented in **Table 3**. Here, we summarize results from the multivariable logistic regression model without social needs. Increasing age was associated with a lower likelihood of readmission [aOR (95% CI): 0.995 (0.993-0.998)]. Patients of non-Hispanic Black or other/unknown race/ethnicity had lower odds of being readmitted than patients of non-Hispanic White race/ethnicity [aOR (95% CI): 0.888 (0.833-0.946) and 0.715 (0.604-0.846), respectively]. Patients with Medicare or Medicaid were significantly more likely to be readmitted than those covered by private insurance [aOR (95% CI): 1.133 (1.043-1.232) and 1.315 (1.182-1.464), respectively]. While there was no significant difference in readmission between type 1 and type 2 diabetes, patients with unknown diabetes had higher odds of readmission than patients with type 2 diabetes [aOR (95% CI): 1.550 (1.212-1.981)].

**Table 3:** Logistic regression models for all-cause 30-day readmissions.

Risk factors	Multivariable logistic regression model without social needs aOR (95% CI) N=26,332 Hospitalizations=42,126	Multivariable logistic regression model with social needs aOR (95% CI) N=26,332 Hospitalizations =42,126
Age	0.995 (0.993-0.998)*	0.996 (0.992-0.999)*
Race/Ethnicity		
Non-Hispanic White	(ref.)	(ref.)
Non-Hispanic Black	0.888 (0.833-0.946)*	0.875 (0.808-0.948)*
Non-Hispanic Asian	0.913 (0.755-1.105)	0.872 (0.714-1.065)
Hispanic	0.894 (0.705-1.133)	0.869 (0.662-1.141)
Other/unknown	0.715 (0.604-0.846)*	0.700 (0.584-0.840)*
Marital status		
Married/Life partner	(ref.)	(ref.)
Single	0.985 (0.913-1.062)	0.982 (0.897-1.075)
Widowed	0.999 (0.911-1.096)	1.037 (0.930-1.157)
Divorced	1.075 (0.984-1.174)	1.067 (0.960-1.186)
Separated	1.096 (0.916-1.312)	1.144 (0.937-1.397)
Insurance		
Medicare	1.133 (1.043-1.232)*	1.098 (0.957-1.261)
Private	(ref.)	(ref.)
Medicaid	1.315 (1.182-1.464)*	1.225 (1.054-1.425)*
Self-pay	0.960 (0.818-1.126)	0.933 (0.765-1.138)
Other/unknown	0.940 (0.771-1.146)	0.943 (0.747-1.191)
VA/Tricare	0.958 (0.749-1.226)	1.193 (0.694-2.052)
Indigent/charity care	1.074 (0.813-1.419)	1.200 (0.832-1.729)
Diabetes type		
Type 2	(ref.)	(ref.)
Type 1	0.925 (0.810-1.056)	0.958 (0.813-1.128)
Unknown	1.550 (1.212-1.981)*	1.485 (1.078-2.046)*
Admission type		
Emergency	(ref.)	(ref.)
Elective	0.791 (0.729-0.858)*	0.785 (0.714-0.863)*
Urgent	1.027 (0.947-1.114)	1.013 (0.926-1.107)
Trauma	0.553 (0.441-0.693)*	0.569 (0.450-0.720)*
Discharge status		
Home	(ref.)	(ref.)
Home health	1.201 (1.126-1.282)*	1.198 (1.111-1.291)*
Skilled nursing facility	1.201 (1.086-1.329)*	1.158 (0.998-1.342)
Against medical advice	1.297 (0.956-1.758)	1.338 (0.953-1.878)
Other	0.818 (0.652-1.026)	0.717 (0.530-0.970)*
Body mass index		
Underweight (<18.5 kg/m^2^)	1.116 (0.937-1.330)	1.094 (0.882-1.357)
Healthy weight (18.5-24.9 kg/m^2^)	1.083 (1.002-1.170)*	1.067 (0.976-1.166)
Overweight (25.0-29.9 kg/m^2^)	1.029 (0.960-1.103)	1.036 (0.963-1.113)
Obese (≥30 kg/m^2^)	(ref.)	(ref.)
Systolic blood pressure		
Normal/elevated (<130 mmHg)	(ref.)	(ref.)
Stage 1 HTN (130-139 mmHg)	0.955 (0.878-1.038)	0.959 (0.874-1.052)
Stage 2 HTN (≥140 mmHg)	0.896 (0.840-0.955)*	0.888 (0.828-0.954)*
Diastolic blood pressure		
Normal (<80 mmHg)	(ref.)	(ref.)
Stage 1 HTN (80-89 mmHg)	0.923 (0.857-0.995)*	0.954 (0.862-1.056)
Stage 2 HTN (≥90 mmHg)	1.092 (1.005-1.188)*	1.116 (1.012-1.231)*
HbA1c (%)	0.985 (0.967-1.004)	0.989 (0.970-1.009)
Albumin (gm/dL)	0.867 (0.823-0.913)*	0.882 (0.826-0.941)*
Creatinine (mg/dL)	0.996 (0.981-1.011)	0.997 (0.978-1.016)
Highest blood glucose (mg/dL)	1.001 (1.000-1.001)*	1.000 (1.000-1.001)*
Lowest blood glucose (mg/dL)	0.999 (0.998-1.000)*	0.999 (0.998-1.000)*
Hematocrit (%)	0.985 (0.980-0.990)*	0.984 (0.979-0.989)*
White blood cell count (10^3^/cmm)	1.004 (1.001-1.007)*	1.003 (1.000-1.007)
Potassium (mMol/L)	1.013 (0.970-1.058)	1.016 (0.966-1.069)
Sodium (mMol/L)	0.994 (0.987-1.001)	0.994 (0.986-1.002)
Length of stay (days)	1.006 (1.003-1.009)*	1.006 (1.003-1.009)*
Charlson Comorbidity Index	1.056 (1.047-1.064)*	1.052 (1.043-1.062)*
Microvascular complications		
0	(ref.)	(ref.)
1	1.040 (0.973-1.111)	1.022 (0.947-1.104)
2	1.031 (0.936-1.136)	1.036 (0.934-1.150)
3	1.109 (0.945-1.302)	1.211 (0.978-1.499)
Anemia diagnosis		
No	(ref.)	(ref.)
Yes	1.125 (1.052-1.204)*	1.125 (1.044-1.213)*
Preadmission insulin use		
No	(ref.)	(ref.)
Yes	1.192 (1.118-1.270)*	1.219 (1.130-1.315)*
Preadmission metformin use		
No	(ref.)	(ref.)
Yes	0.921 (0.847-1.001)	0.924 (0.845-1.010)
Preadmission GLP-1 use		
No	(ref.)	(ref.)
Yes	0.923 (0.756-1.126)	1.045 (0.805-1.356)
Preadmission SGLT2 use		
No	(ref.)	(ref.)
Yes	1.054 (0.812-1.370)	1.189 (0.857-1.648)
Preadmission TZD use		
No	(ref.)	(ref.)
Yes	0.728 (0.506-1.048)	0.755 (0.504-1.131)
Prior admission within 90 days of index hospital admission		
No	(ref.)	(ref.)
Yes	1.149 (1.023-1.289)*	1.169 (1.032-1.323)*
Discharge status of most recent hospital stay within last year		
No hospitalization within past year	(ref.)	(ref.)
Home	1.504 (1.367-1.655)*	1.448 (1.302-1.611)*
Home health	1.438 (1.276-1.620)*	1.381 (1.200-1.589)*
Skilled nursing facility	1.353 (1.090-1.680)*	1.166 (0.847-1.604)
Against medical advice	2.206 (1.256-3.847)*	2.025 (1.121-3.660)*
Other	1.119 (0.918-1.364)	1.036 (0.827-1.298)
Follow-up appointment after discharge		
Yes	(ref.)	(ref.)
No	0.927 (0.873-0.985)*	0.934 (0.871-1.002)
Activities of daily living		
Independent	-	(ref.)
Needs some help	-	0.986 (0.851-1.143)
Dependent	-	1.141 (0.854-1.523)
Alcohol use		
Current	-	0.947 (0.839-1.069)
Former	-	1.114 (0.995-1.247)
None	-	(ref.)
Within the past year	-	1.048 (0.814-1.351)
Substance use		
Current	-	1.013 (0.806-1.273)
Former	-	1.047 (0.886-1.236)
None	-	(ref.)
Within the past year	-	1.054 (0.726-1.531)
Smoking/tobacco use		
Current smoker	-	1.022 (0.903-1.156)
Former smoker	-	1.027 (0.935-1.129)
Never smoked	-	(ref.)
Employment status		
Retired	-	1.045 (0.860-1.269)
Disabled	-	1.100 (0.917-1.319)
Employed	-	(ref.)
Unemployed	-	1.006 (0.783-1.292)
Others (part time or student)	-	1.048 (0.725-1.513)
Housing stability		
Home	-	(ref.)
Unstable housing	-	1.147 (0.777-1.694)
Homeless	-	1.019 (0.773-1.345)
Others	-	1.046 (0.793-1.380)
Social support		
Yes	-	(ref.)
No	-	0.970 (0.722-1.305)
Stressors		
Health-related stressor	-	(ref.)
Finances	-	1.132 (0.674-1.901)
Social stressor	-	0.685 (0.383-1.227)

*Statistical significance at p<0.05.

aOR, adjusted odds ratio; VA, Veterans Administration; HTN, hypertension; GLP-1, glucagon-like peptide-1 receptor agonists; TZD, thiazolidinediones; SGLT2, sodium–glucose cotransporter-2 inhibitors.

Index hospitalizations classified as elective or trauma admissions had lower odds of being readmitted than those with emergency-related admissions [aOR (95% CI): 0.791 (0.729-0.858) and 0.553 (0.441-0.693), respectively]. Index hospitalizations discharged to home health or skilled nursing facilities had higher odds of being readmitted than those discharged to home [aOR (95% CI): 1.201 (1.126-1.282) and 1.201 (1.086-1.329), respectively]. The healthy BMI category was associated with higher odds of readmission compared to obese BMI [aOR (95% CI): 1.083 (1.002-1.170)]. Stage 1 hypertension (diastolic blood pressure 80-90 mmHg) was associated with lower odds of readmission compared to normal diastolic blood pressure [aOR (95% CI): 0.923 (0.857-0.995)], but stage 2 hypertension (diastolic blood pressure ≥90 mmHg) was associated with higher odds of readmission [aOR (95% CI): 1.092 (1.005-1.188)].

HbA1c was not significantly associated with readmission status. For blood glucose measurements during hospitalization, higher values for the highest blood glucose were associated with a higher likelihood of readmission, and increasing values for the lowest blood glucose were associated with a lower likelihood of readmission. Other labs, including albumin [aOR (95% CI): 0.867 (0.823-0.913)], hematocrit [aOR (95% CI): 0.985 (0.980-0.990)], and white blood cell count [aOR (95% CI): 1.004 (1.001-1.007)], were significantly associated with readmission status. The odds of readmission were increased with a longer length of stay [aOR (95% CI): 1.006 (1.003-1.009)] and a higher CCI [aOR (95% CI): 1.056 (1.047-1.064)].

An anemia diagnosis increased the odds of readmission compared to no diagnosis [aOR (95% CI): 1.125 (1.052-1.204)]. Preadmission insulin use increased the odds of readmission [aOR (95% CI): 1.192 (1.118-1.270)], but other diabetes medication classes were not associated with readmission status. A prior hospital stay within the past 90 days increased the odds of readmission [aOR (95% CI): 1.149 (1.023-1.289)]. The discharge status of the most recent hospitalization within the last year was a significant predictor of readmission. Discharges to home [aOR (95% CI): 1.504 (1.367-1.655)], home health [aOR (95% CI): 1.438 (1.276-1.620)], skilled nursing facility [aOR (95% CI): 1.353 (1.090-1.680)], or against medical advice [aOR (95% CI): 2.206 (1.256-3.847)] were associated with increased odds of readmission compared to those with no prior hospitalizations within the last year. Lastly, no follow-up appointment after discharge was associated with lower odds of readmission [aOR (95% CI): 0.927 (0.873-0.985)].

### Added predictive value of social needs

We briefly highlight the different results for the regression model with social needs here. The risk factors no longer significantly associated with readmission status were Medicare versus private insurance, discharge status to a skilled nursing facility, healthy BMI category, diastolic blood pressure 80-89 mmHg, white blood cell count, discharge status of most recent hospital stay to a skilled nursing facility, and follow-up appointment after discharge. After controlling for clinical risk factors in the baseline model and social needs, index hospital discharge status to other vs. home was significantly associated with a lower likelihood of readmission [aOR (95% CI): 0.717 (0.530-0.970)]. Here, no social needs were significantly associated with readmission status (p≥0.05; see **Table 3** for aORs for social needs). In the sensitivity analysis, we excluded social needs with high levels of missingness (≥70%) from the regression model (i.e., activities of daily living, social support, and types of stressors). Results were similar to the two reported regression models in **Table 3**, except for the result for former versus no alcohol use. In the sensitivity analysis, former alcohol use was significantly associated with higher odds of readmission compared to no alcohol use [aOR (95% CI): 1.121 (1.008-1.247)].

## Discussion

We identified various factors associated with 30-day readmissions among patients with diabetes in the Deep South. These factors included demographics (i.e., age, race/ethnicity, insurance status, unknown diabetes type), characteristics of hospitalizations (i.e., admission type, discharge status, length of stay, prior hospitalizations, discharge status of the most recent hospital stay within the last year), labs and vitals (i.e., albumin, hematocrit, highest and lowest blood glucose measurements, blood pressure), co-existing chronic conditions (i.e., CCI score and anemia diagnosis), preadmission medication use (i.e., insulin use), and social need (i.e., former alcohol use). These risk factors can support the readmission risk assessment for patients with diabetes in the Deep South. Factors associated with readmission risk can help identify high-risk patient groups for all-cause 30-day readmissions during pharmacy clinical services.

Our work in studying readmission risk among patients with diabetes in the Deep South expands upon the foundational work by Rubin et al. and Karunakaran et al. in creating, validating, and extending the DERRI™ (13–15). Our findings apply these prior works to the Deep South population. The majority of risk factors we found to be associated with readmission risk are supported by similar findings from Karunakaran et al., but some key differences were found for risk factors, such as age, gender, employment status, creatinine, having a follow-up appointment after discharge, etc. (13). Looking at pooled results across studies from a systematic review, our findings for readmission risk being associated with insurance type, comorbidities, insulin use, and length of stay align closely with prior literature (11). However, our findings for gender, race, and age contrasted with their results, demonstrating key differences in findings from the Deep South population compared to other U.S. populations (11).

Our study adds value and new information to the transitions of care literature in its comprehensive assessment of factors influencing readmissions among people with diabetes and its expansion to include social needs. Even though social factors have long been recognized to influence health outcomes (18), limited studies investigating readmissions have considered social needs among non-disease-specific populations (20, 21). In contrast, a recent study by Pinheiro et al. recognized the cumulative effect of social needs in increasing patients’ risk for heart failure-related hospitalizations (36). Our study applied a similar approach to Pinheiro et al. (36) by applying their methods to the diabetes context investigating the influence of social needs on readmissions. We identified various social needs associated with readmission risk in unadjusted analyses, and we found former alcohol use to be associated with an increased risk of readmission in the sensitivity analysis. Thus, alcohol use was found to be “an independent predictor” (34) of 30-day readmissions among patients with diabetes in the Deep South. Prior literature among other populations also found alcohol use/abuse to increase readmission risk (37) or to have no significant effect (38). We are limited in interpreting our finding of the potential relationship between alcohol use and readmission because all people reporting alcohol use were grouped together into “current” or “former” alcohol use categories. Thus, we were not able to determine the amount of alcohol consumption or to differentiate between alcohol use versus abuse, which could influence the relationship with readmissions.

The recognized importance and consideration of social needs in health outcomes and clinical care also bring forth expected challenges. Our experience through this study demonstrates the challenges from incomplete data capture of social needs within EHRs. The missingness of data for social needs varied by concept, ranging from a low of 40% for smoking/tobacco use to a high of 98% for type of stressors. Due to the lack of complete data, we imputed missing data using multiple imputation. Imputation of social needs with high missingness likely limited our ability to learn anything about the associations we were interested in testing due to high variance. Thus, the high levels of missingness for social needs may have influenced the nonsignificant findings between social needs and readmission status in the adjusted regression model. Still, our findings of significant relationships between social needs and readmission status in either unadjusted or the sensitivity analysis support a call for further research. Further research with more complete social needs data is needed to understand the influence of social needs on readmissions among populations with diabetes. Recent work in linking EHRs with social factors available through U.S. Census data brings a potential solution to the challenge of capturing social needs in clinical data (39). Further work is also needed to understand the potential clinical utility of incorporating social needs into clinical services. For instance, pharmacy clinical services could serve an integral role in collecting social needs data from patients given pharmacists’ more routine interactions with patients compared to other healthcare settings.

One major strength of this study and its findings is its focus on patients with diabetes in the Deep South. Our focus on the Deep South fills the existing research gap for readmissions among the diabetes population in this area, where the prevalence of diabetes surpasses the national average (22). Prior literature in this realm has studied many other populations with diabetes, such as insured populations (commercial, Medicare, or Medicaid), clinical data from the general U.S. population, or EHRs from health systems in the Northeast (9, 11–15, 40). In the present study, we demonstrated the multitude of risk factors contributing to readmission risk among patients with diabetes in the Deep South. Our findings can support decision-making around factors influencing patients’ readmission risks in the Deep South, including expanding healthcare decision-making to consider individuals’ social needs (41). Our findings can also provide evidence for future intervention studies for populations with diabetes. Different clinical interventions, including pharmacy clinical services (42), have been efficacious in reducing hospital readmission rates among patients with diabetes (5, 43). Community health workers could serve a vital role in coordinating social needs through community-based interventions to further reduce readmissions (44). Further, incorporating social needs into a case management intervention has proven beneficial in reducing inpatient admissions (45).

Another key strength of this study was investigating readmission risk by diabetes type. We found no significant difference in readmission risk for patients diagnosed with type 1 versus type 2 diabetes. Through a recent systematic review, Soh et al. identified a gap in the literature studying readmission risk by diabetes type because most studies lump patients with type 1 and type 2 diabetes together (11). To address this gap, we included diabetes type as a risk factor in studying readmission risk. Our findings demonstrate that diabetes type may not significantly affect readmission risk among patients in the Deep South. However, patients with unknown diabetes type had significantly higher odds of 30-day readmission than patients with type 2 diabetes. Patients were classified as having an unknown diabetes type because they were prescribed a diabetes medication before the hospitalization but did not have any diabetes diagnosis codes. The higher readmission risk for patients with unknown diabetes in the Deep South is an interesting finding that calls for further investigation. Our finding is supported by prior research showing a higher likelihood of readmission when diabetes is not coded in the medical record, further highlighting the importance of diabetes even when patients may be hospitalized for other reasons (40).

In conclusion, we identified the factors that impacted the risk of 30-day readmissions among patients with diabetes in the Deep South. Clinical assessment of readmission risk in the Deep South should consider patients’ demographics, characteristics of hospitalizations, labs, vitals, co-existing chronic conditions, preadmission antihyperglycemic medication use, and social needs. Factors associated with readmission risk can help pharmacists and other healthcare providers identify high-risk patient groups for all-cause 30-day readmissions during transitions of care. Further research is needed about the influence of social needs on readmissions among populations with diabetes to understand the potential clinical utility of incorporating social needs into clinical services.

## Limitations

We cautiously report that factors are associated with increased readmission risk due to the secondary data analysis of EHRs. Because our data source was EHRs, we were limited to capturing readmissions within our health system, and we may have missed readmissions occurring in another health system. Although we made efforts to control for confounding variables in the adjusted analysis, there is still potential for residual confounding. We were also limited in analyzing factors available in the EHRs. Other factors that might be expected to influence readmissions and diabetes care, such as detailed information from physician notes about discharge planning, self-care behaviors, psychosocial factors (e.g., diabetes distress) (46), or other social needs (38), were not available in the limited dataset. The missingness of social needs data is a major limitation of this study, and the population of patients reporting social needs could represent a biased sample. Clinically important variables, such as cholesterol and procedures, were not available in the data source. We also did not have access to 9-digit zip codes, which prevented us from calculating the patients’ living distance from the hospital that has been continually documented as an important predictor for readmission in patients with diabetes (13–15). We acknowledge that this data was collected for clinical practice purposes rather than research purposes, so the reliability of data elements, such as outpatient prescription records, could be a limitation. However, previous research among patients with diabetes has shown that prescription orders documented in EHRs can be used to represent prescriptions filled and dispensed (47). Lastly, there is a potential for misclassification bias in identifying patients with diabetes. Patients taking antihyperglycemic medications for other conditions (e.g., metformin for prediabetes or polycystic ovary syndrome) could have been incorrectly classified as having diabetes. However, this was not expected to have major effects on findings because less than 0.9% of patients took antihyperglycemic medications but did not have a diabetes diagnosis code.

## Data availability statement

The data analyzed in this study is subject to the following licenses/restrictions: The dataset used in this study is confidential and cannot be shared.

## Ethics statement

The studies involving human participants were reviewed and approved by Auburn University Institutional Review Board for the Protection of Human Subjects in Research (IRB). Written informed consent for participation was not required for this study in accordance with the national legislation and the institutional requirements.

## Author contributions

CM and CC contributed to conceptualization, methodology, data acquisition, investigation, project administration, funding acquisition, and reviewing/editing the manuscript. CM performed software programming, data curation, formal analysis, and writing the original manuscript draft. CC supervised the research. All authors contributed to the article and approved the submitted version.

## Funding

This study was supported by the National Center for Advancing Translational Sciences of the National Institutes of Health under award number TL1TR003106. The content is solely the responsibility of the authors and does not necessarily represent the official views of the National Institutes of Health. McDaniel was supported by the American Foundation for Pharmaceutical Education (AFPE), and she is currently supported by the PhRMA Foundation under the Pre-Doctoral Fellowship in Health Outcomes Research. Chou is currently supported by the PhRMA Collaborative Actions to Reach Equity (CAREs) grant program.

## Acknowledgments

Preliminary results from this work were presented in abstract form at the Association for Clinical and Translational Science Annual Meeting in April 2021 and the American Association of Colleges of Pharmacy Annual Meeting in July 2022.

## Conflict of interest

The authors declare that the research was conducted in the absence of any commercial or financial relationships that could be construed as a potential conflict of interest.

## Publisher’s note

All claims expressed in this article are solely those of the authors and do not necessarily represent those of their affiliated organizations, or those of the publisher, the editors and the reviewers. Any product that may be evaluated in this article, or claim that may be made by its manufacturer, is not guaranteed or endorsed by the publisher.
