# Supplementary material for: Clinical risk factors and social needs of 30-day readmission among patients with diabetes: A retrospective study of the Deep South
Source: Front Clin Diabetes Healthc. 2022 Oct 26;3:1050579. doi: 10.3389/fcdhc.2022.1050579 (PMC10012098; doi:10.3389/fcdhc.2022.1050579)
Supplement: Supplementary file 1 [file Table_1.docx]

Supplementary Material

Supplemental Table. Risk factors details

| **Risk factor** | **Level of measurement** | **Period of measurement** |
| --- | --- | --- |
| Age | Continuous | During index hospitalization* |
| Race/Ethnicity | Nominal | During index hospitalization* |
| Gender | Nominal | During index hospitalization* |
| Marital status | Nominal | During index hospitalization* |
| Insurance | Nominal | During index hospitalization* |
| Diabetes type | Nominal | Any time prior to index hospitalization |
| Admission type | Nominal | During index hospitalization* |
| Discharge status | Nominal | During index hospitalization* |
| Body mass index | Ordinal (following clinical cut-points) | 6 months pre-index through discharge date of index hospitalization |
| Systolic blood pressure | Ordinal (following clinical cut-points) | 6 months pre-index through discharge date of index hospitalization |
| Diastolic blood pressure | Ordinal (following clinical cut-points) | 6 months pre-index through discharge date of index hospitalization |
| HbA1c (%) | Continuous | 6 months pre-index through discharge date of index hospitalization |
| Albumin (gm/dL) | Continuous | 6 months pre-index through discharge date of index hospitalization |
| Creatinine (mg/dL) | Continuous | 6 months pre-index through discharge date of index hospitalization |
| Highest blood glucose (mg/dL) | Continuous | During index hospitalization* |
| Lowest blood glucose (mg/dL) | Continuous | During index hospitalization* |
| Hematocrit (%) | Continuous | 6 months pre-index through discharge date of index hospitalization |
| White blood cell count (10^3^/cmm) | Continuous | 6 months pre-index through discharge date of index hospitalization |
| Potassium (mMol/L), | Continuous | 6 months pre-index through discharge date of index hospitalization |
| Sodium (mMol/L) | Continuous | 6 months pre-index through discharge date of index hospitalization |
| Length of stay (days) | Continuous | During index hospitalization* |
| Charlson Comorbidity Index | Continuous | 6 months pre-index through discharge date of index hospitalization |
| Macrovascular complications | Ordinal (0-4) | 6 months pre-index through discharge date of index hospitalization |
| Microvascular complications | Ordinal (0-3) | 6 months pre-index through discharge date of index hospitalization |
| Anemia diagnosis | Nominal | 6 months pre-index through discharge date of index hospitalization |
| Preadmission insulin use | Nominal | 6 months pre-index |
| Preadmission metformin use | Nominal | 6 months pre-index |
| Preadmission sulfonylurea use | Nominal | 6 months pre-index |
| Preadmission GLP-1 use | Nominal | 6 months pre-index |
| Preadmission DPP-4 use | Nominal | 6 months pre-index |
| Preadmission SGLT2 use | Nominal | 6 months pre-index |
| Preadmission TZD use | Nominal | 6 months pre-index |
| Preadmission other diabetes medications | Nominal | 6 months pre-index |
| Prior admission within 90 days of index hospital admission | Nominal | Specific point in time |
| Discharge status of most recent hospital stay within last year | Nominal | Specific point in time |
| Follow-up appointment after discharge | Nominal | Specific point in time |
| Discharge year | Nominal | During index hospitalization* |
| Social needs | Nominal | 6 months pre-index through discharge date of index hospitalization |

*During index hospitalization (i.e., from the admission date through the discharge date)

Abbreviations: GLP-1=glucagon-like peptide-1 receptor agonists, TZD=thiazolidinediones, DPP-4=dipeptidyl peptidase 4 inhibitors, SGLT2=sodium–glucose cotransporter-2 inhibitors.
